# Contrast-enhanced ultrasound versus conventional ultrasound-guided percutaneous nephrolithotomy in patients with a non-dilated collecting system: results of a pooled analysis of randomized controlled trials

**DOI:** 10.1186/s12894-023-01269-8

**Published:** 2023-05-12

**Authors:** Li Wang, Kun-peng Li, Shan Yin, Lin Yang, Ping-yu Zhu

**Affiliations:** 1 Department of Urology, Affiliated Hospital of North Sichuan Medical College, Nanchong, China; 2 Department of Urology, The Second Hospital of Lanzhou University, Lanzhou, China

**Keywords:** Contrast-enhanced ultrasound, Percutaneous nephrolithotomy, Urinary calculi, Urolithiasis

## Abstract

**Background:**

Contrast-enhanced ultrasound in percutaneous nephrolithotomy (CEUS-PCNL) is an economical and practical technique for the treatment of patients with renal stones without significant collecting system dilatation. The aim of this systematic review is to compare the safety and efficacy of CEUS-PCNL and conventional ultrasound (US)-guided (US-PCNL) treatment of patients with renal calculi without significant hydronephrosis.

**Methods:**

This review was conducted with strict adherence to the PRISMA guidelines. Comparative studies on CEUS-PCNL and US-PCNL published in PubMed, SinoMed, Google Scholar, Embase, and Web of science until March 1, 2023, were systematically searched. RevMan 5.1 software was used for meta-analysis. Pooled odds ratios (ORs), weight mean differences (WMDs) and standard mean differences (SMDs) with 95% confidence intervals (CIs) were calculated using the fixed-effects or random-effects model. Publication bias was evaluated using funnel plots.

**Results:**

Four randomized controlled trials involving 334 patients (168 with CEUS-guided PCNL and 166 with US-guided PCNL) were identified. There was no statistically significant difference between CEUS-guided PCNL and US-guided PCNL in terms of the operation time (SMD: − 0.14; 95% CI − 0.35 to 0.08; p = 0.21), minor complications (p = 0.48), major complications (p = 0.28) and overall complications (p = 0.25). However, CEUS-guided PCNL had a higher stone-free rate (OR: 2.22; 95% CI 1.2 to 4.12; p = 0.01), higher success rate of single-needle punctures (OR:3.29; 95% CI 1.82 to 5.95; p < 0.0001), shorter puncture time (SMD: − 1.35; 95% CI − 1.9 to − 0.79; p < 0.00001), shorter hospital stay (SMD: − 0.34; 95% CI − 0.55 to − 0.12; p = 0.002) and lesser hemoglobin loss (SMD: − 0.83; 95% CI − 1.06 to − 0.61; p < 0.00001) as compared with conventional US-guided PCNL.

**Conclusions:**

According to almost all pooled data, CEUS-guided PCNL is superior to US-guided PCNL in terms of the perioperative outcomes. However, many rigorous clinical randomized controlled studies are required to obtain more accurate results.

*Registration* The study protocol was registered with PROSPERO (CRD42022367060).

**Supplementary Information:**

The online version contains supplementary material available at 10.1186/s12894-023-01269-8.

## Introduction

Urolithiasis is one of the most prevalent diseases of the urinary system, and its incidence continues to increase every year [1]. According to the American Urological Association, most patients with staghorn renal stones and more giant kidney stones (> 20 mm) should be recommended percutaneous nephrolithotomy (PCNL) as their first treatment option [2]. Compared with other open surgical methods, PCNL has a more efficient stone clearance rate and is less invasive; However, PCNL has a potential for serious complications, such as bleeding and injury to peripheral organs. Consequently, precise needle puncture of the kidney is the most critical stage in PCNL surgery [3, 4].

Fluoroscopic guidance, traditionally used for renal access, can accurately identify the calyx to be punctured. A significant disadvantage of fluoroscopic guidance is its inability to visualize adjacent organs in real time, which increases the risk of damaging surrounding structures. Additionally, exposure to ionizing radiation emitted by this method can harm patients and healthcare professionals [5]. By contrast, ultrasound-guided renal puncture has effectively demonstrated efficacy and safety as an alternative to X-ray guidance, reducing radiation hazards while providing real-time guidance.

However, for young physicians, the learning curve of ultrasound-guided PCNL is more prolonged, especially when dealing with an unexpanded renal collecting system, and the degree of calyceal puncture visualization is low. Clinical practice has found that using large amounts of saline to create artificial hydronephrosis causes the kidneys to gradually become pale and blurred on ultrasound. This can decrease the surgery success rate, and increase the postoperative complications [6].

According to a single-centre retrospective investigation conducted by Liu et al. [7], contrast-enhanced US-assisted PCNL is beneficial for patients with a nondilated renal collecting system, and is accompanied by a high success rate and tolerable sequelae. Recent investigations have been compared contrast-enhanced ultrasound with traditional ultrasound-guided PCNL [8–11]. We performed this meta-analysis to assess the effectiveness and security of these two various surgically assisted ultrasound-guided techniques.

## Methods

### Search strategy

The search strategies, selection criteria and evidence report were formulated according to the recommendations of Preferred Reporting Items for Systematic Review and Meta-Analysis (PRISMA) (Additional file 1: Table S1), and the study was registered on the PROSPERO database (ID: CRD42022367060).

The Cochrane Library, PubMed, SinoMed, Google Scholar, Embase and Web of Science databases were comprehensively searched for relevant studies published until March 1, 2023. The titles and abstracts were preliminarily assessed, after which the full texts of the relevant studies were acquired. The reference lists of the selected articles were also scrutinized for any extra potential studies. The following search string was constructed by combining the terms related to patients and interventions: [(Urinary calculi OR Kidney stone OR Renal calculi OR Urolithiasis) AND (Contrast-enhanced ultrasound OR Ultrasound OR Ultrasonic contrast) AND (Percutaneous nephrolithotomy OR Percutaneous)].

No language-based limitations were imposed.

### Inclusion and exclusion criteria

To determine the studies to be included, the abovementioned search strategy was constructed in accordance with the PICOS framework. [P (patients): patients with non-dilated collecting system kidney stones who required PCNL surgery; I (intervention): CEUS-guide PCNL performed; C (comparator): conventional US-guided PCNL used as a comparison; O (outcomes): perioperative outcomes and complications; S (study type): prospective and retrospective case–control studies as well as randomized controlled trials (RCTs)]. The exclusion criteria were as follows: (1) no relevant data available for meta‐analysis; (2) non-comparative studies and (3) conference abstracts, case reports, letters and any other unpublished articles.

### Screening process and data extraction

Using Endnote X9 (London, UK), two reviewers (WL and KP) distinguished the conclusive literature by eliminating duplicates, perusing title-level abstracts and performing a full-text audit based on the set inclusion and exclusion criteria for all the incorporated studies. A senior researcher (YS) was consulted in case of disparities. The data extraction process was subsequently implemented, trailed by the ordering of the study data using pre-set Excel tables.

Two reviewers (WL and YL) independently extracted the following details: first author name, year of publication, type of study, country, number of patients, age, body mass index (BMI), gender, PCNL position, lithotripsy technique, CEUS guided PCNL technique, contrast agent used, stone characteristics and follow-up. Furthermore, the following outcomes were retrieved: perioperative outcomes including puncture time, the stone-free rate (SFR), decrease in hemoglobin levels, hospital stay duration, operation time, single-needle puncture success rate, and postoperative complications.

### Risk of bias and quality assessment

The Cochrane Handbook for Systematic Reviews of Interventions was used to assess the risk of bias [12]. The assessment comprised the analysis of selection bias, attrition bias, blinding and sample size. For all the studies included in this meta-analysis, the level of evidence (LE) was independently estimated according to the criteria provided by the Oxford Centre for Evidence-Based Medicine [13]. The evaluation was completed by two separate reviewers (KP and YS), and any discrepancies were resolved through negotiation.

### Statistical analysis

Review Manager 5.3 (Cochrane Collaboration, Oxford, UK) was used for the meta-analysis [14]. Odds ratios (ORs) with 95% confidence intervals (CIs) was used for dichotomous variables. Standard mean differences (SMDs) and Weight mean differences (WMDs) with 95% confidence intervals (CIs) were used to present continuous variables. McGrath et al.’s formula was utilized to translate the median and range of data for the means and standard deviations [15]. If there was significant heterogeneity at I^2^ > 50%, random-effect models were selected according to the Cochrane review principles. In all other situations, fixed-effect models were applied. P < 0.05 was considered statistically significant.

### Sensitivity analyses and publication bias

Potential sources of heterogeneity, if significant, were explored using sensitivity analysis, performed using leave-one-out methods. However, this condition was not applied when the comparison involved fewer than three studies [16].

Because of the insufficient test power when fewer than 10 studies are included, linear regression-based publication bias detection methods, such as the Begg and Egger tests, may be inaccurate [17]. Thus, we used the visual symmetry of the funnel plot to roughly estimate the publication bias.

## Results

### Baseline characteristics

**Fig. 1:**
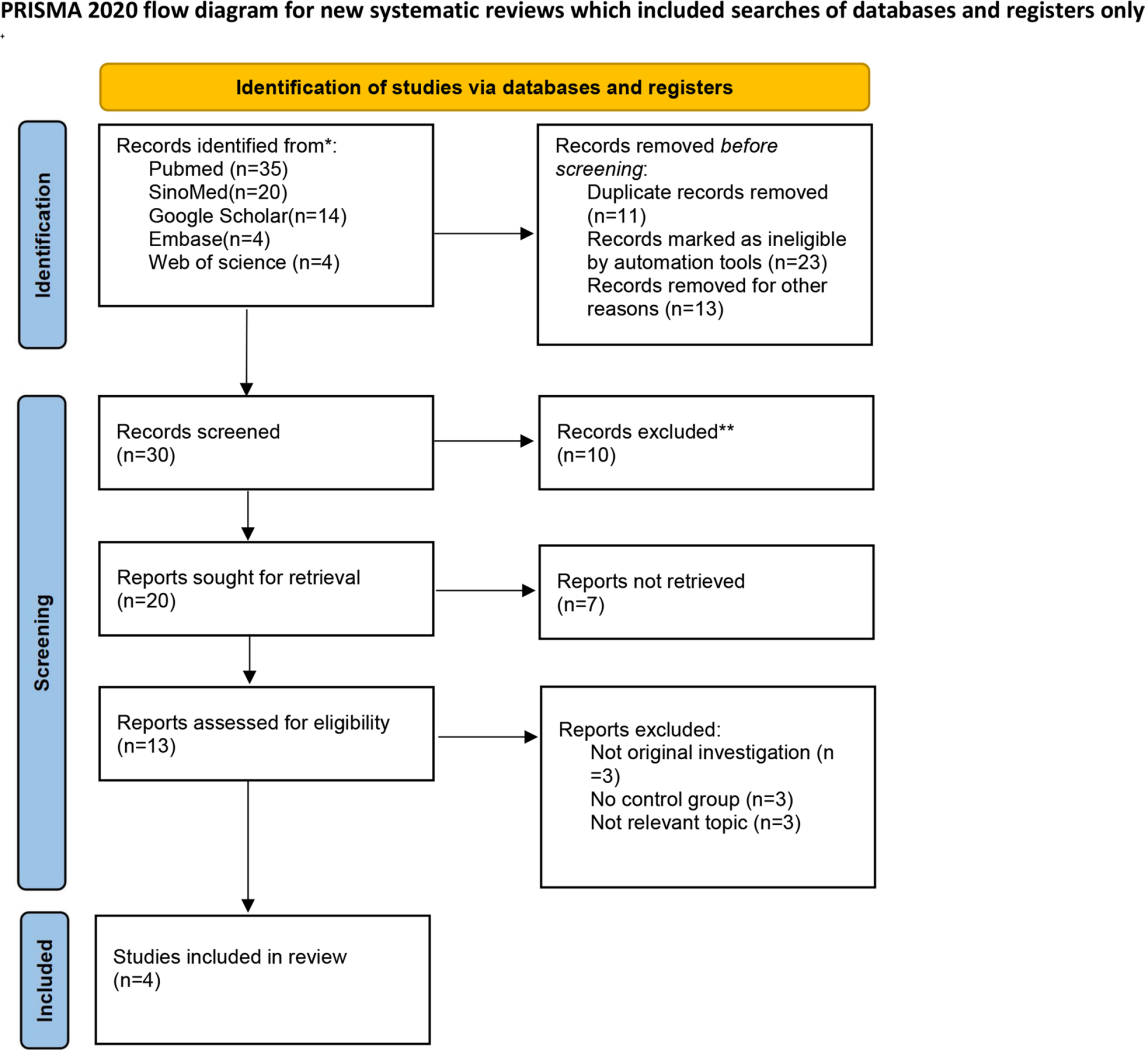
PRISMA 2020 flow diagram for the systematic review. *Consider, if feasible to do so, reporting the number of records identified from each database or register searched (rather than the total number across all databases/register). ** If automation tools were used, indicate how many records were excluded by a human and how many were excluded by automation tools.

The search design tactics yielded a total of 77 records. After eliminating the duplicates and reviewing the titles, abstracts and full texts, four randomized controlled studies [8–11, 18] involving 328 patients (168 in the CEUS group and 166 in the conventional US group) were included in the study. The literature screening process is shown in (Fig. 1).

The key characteristics of the patients in the collected studies are shown in Table 1. Table 2 shows the related characteristics of the urinary calculi. All of the RCTs we found explained their randomization process. There were no significant differences in the age (p = 0.1), BMI (p = 0.06), gender (male) (p = 0.16), stone laterality (p = 0.91) and stone size (p = 0.45) between the CEUS-PCNL and US-PCNL groups (Table 3).

**Table 1:** Baseline characteristics of enrolled trials

Study	Country	Study design	PCNL patients		PCNL position	Lithotripsy technique	Outcomes	CEUS-PCNL technique	Contrast agent using	Follow-up time (day)	LE
			CEUS-guided	US-guided							
Guo (2020) [8]	China	RCT	30	30	Prone	Holmium laser (Lumenis 60W, Germany)	1,2,3,4,5,6,7	After general anesthesia and prophylactic use of antibiotics, each patient was placed in the lithotomy position. An open-ended 5-F ureteral catheter was placed retrograde into the ipsilateral proximal ureter up to 25 cm under ureteroscope guidance. The patient was then placed in the prone position	Sulfur hexafluoride microbubbles (SonoVue; Bracco, Switzerland)	30	2b
Liu (2022) [9]	China	RCT	36	36	Flank	NA	1,2,3,4,5,6,7	Under general anesthesia and with the patient supine, an open-ended 6-F ureteral catheter was retrograde placed into the ipsilateral proximal ureter up to 25 cm under ureteroscopic guidance. Patients were then moved to a complete lateral decubitus position	Sulfur hexafluoride microbubbles (SonoVue; Bracco, Switzerland)	28	2b
Xia (2021) [10]	China	RCT	78	76	Prone	The laser energy used ranged from 30 to 60W (1.5–3.0 J*20 Hz)	1,2,3,4,5,6,7	Under general anesthesia and with the patient supine, an open-ended 5-F ureteral catheter was advanced retrograde into the ipsilateral proximal ureter up to 25 cm under cystoscopic guidance	Sulfur hexafluoride microbubbles (SonoVue; Bracco, Switzerland)	30	2b
Liu (2021) [11]	China	RCT	24	24	Flank	Holmium laser (LUME.PK)	1,2,4,5,6,7	Under general anesthesia and with the patient supine, an open-ended 6-F ureteral catheter was retrograde placed into the ipsilateral proximal ureter under ureteroscopic guidance	Sulfur hexafluoride microbubbles (SonoVue; Bracco, Switzerland)	7	2b

RCT: randomized controlled trial; US: ultrasonography; CEUS: contrast-enhanced ultrasound; PCNL: percutaneous nephrolithotomy; LE: level of evidence according to the Oxford Center for Evidence-based Medicine; NA: not available

1 = puncture time; 2 = stone-free rate; 3 = complications; 4 = hemoglobin loss; 5 = hospital stay; 6 = operation time; 7 = single-needle puncture success rate

**Table 2:** The specific features of renal stones

Reference	CEUS-guided PCNL/US-guided PCNL				
	Multiple stones, n	Location, n	Degree of hydronephrosis	Right side, n	Mean stone size (mm)
Guo et al. (2020) [8]	NA	Renal pelvic:3/4	NA	11/13	27.6/27.2
		Partial staghorn:14/11			
		complete staghorn:3/4			
		Renal pelvis and calyceal:10/11			
Liu et al. (2022) [9]	NA	Renal pelvic:2/1	None:21/21	17/19	37.6/36.5
		calyceal:3/4	Mild:15/15		
		Renal Pelvis and calyceal:10/10			
		Partial staghorn:8/11			
		complete staghorn:13/10			
Xia et al. (2021) [10]	17/14	Upper calyx:10/12	NA	40/36	23/22
		Middle calyx:11/11			
		Lower calyx:13/15			
		Renal pelvis:26/24			
Liu et al. (2021) [11]	NA	NA	None:15/16	15/15	39.3/35.8
			Mild:9/8		

CEUS: contrast-enhanced ultrasound; US: ultrasound; NA: not available

**Table 3:** Comparison of the patients baseline characteristics

Baseline characteristic	No. of studies	CEUS-guided PCNL versus US-guided PCNL	Heterogeneity I^2^ (%)	p value
Age WMD (95% CI)	4	− 1.87 (− 4.12 to 0.39)	0	0.1
BMI WMD (95% CI)	4	− 0.54 (− 1.11 to 0.03)	0	0.06
Gender (male) OR (95% CI)	4	1.39 (0.87 to 2.22)	0	0.16
Left side OR (95% CI)	4	1.02 (0.66 to 1.58)	0	0.91
Stone size WMD (95% CI)	4	0.08 (− 0.12 to 0.27)	0	0.45

CEUS: contrast-enhanced ultrasound; US: ultrasound; NA: not available; PCNL: percutaneous nephrolithotomy; BMI: body mass index; WMD: weight mean difference; OR: odds ratio; CI: confidence interval

### Methodological quality

**Fig. 2:**
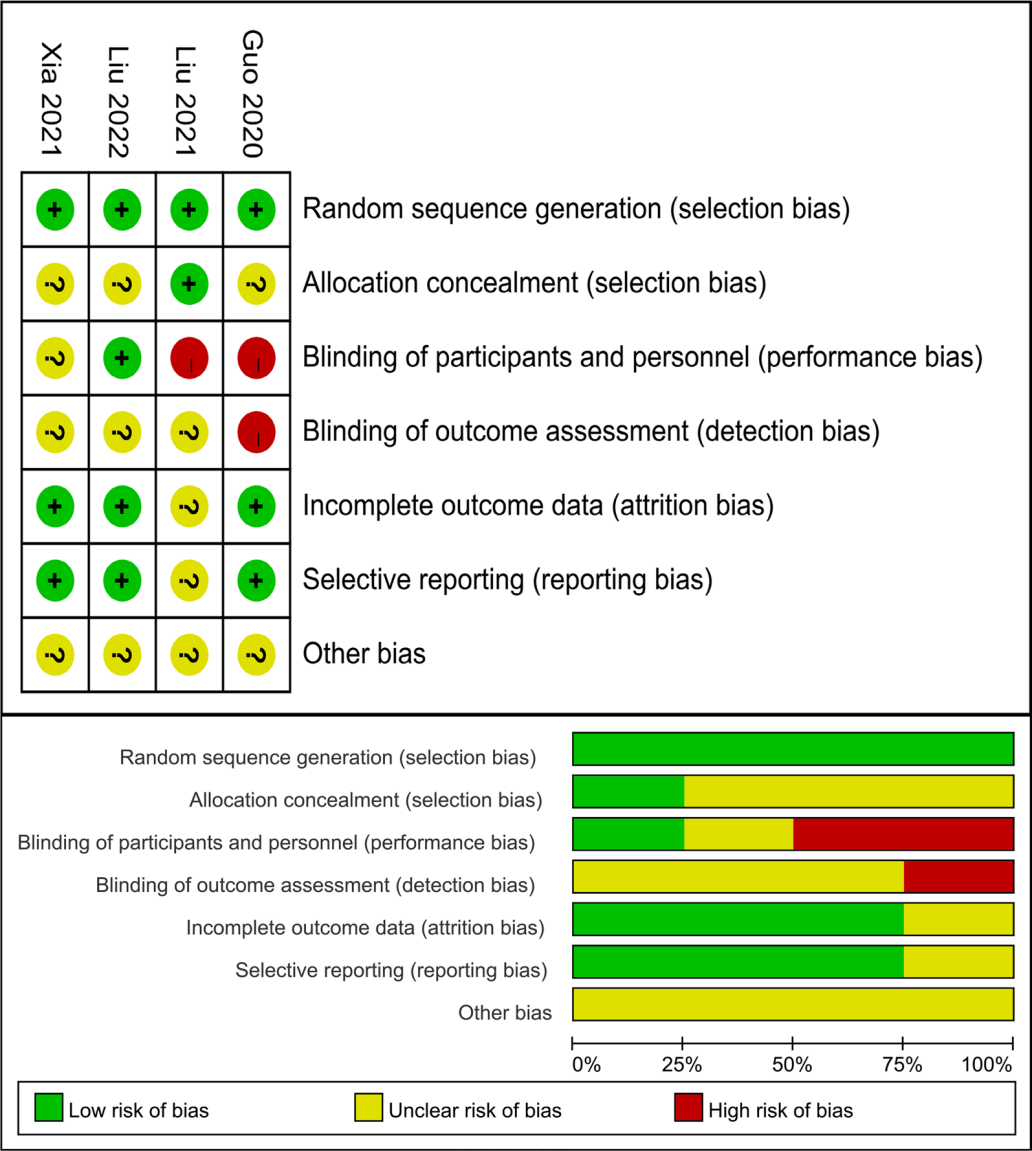
Risk of bias assessment of the included RCTs

The results of the Cochrane risk assessment are shown in (Fig. 2). All investigations were of a high quality overall. there was no concealed assignment in three trials [8–10]. Two studies did not employ double blinding during the process, whereas one trial did not record participant and personnel blinding. The level of evidence was 2b for all the included studies, which was considered appropriate for this meta-analysis (Table 1).

### Perioperative outcomes

**Fig. 3:**
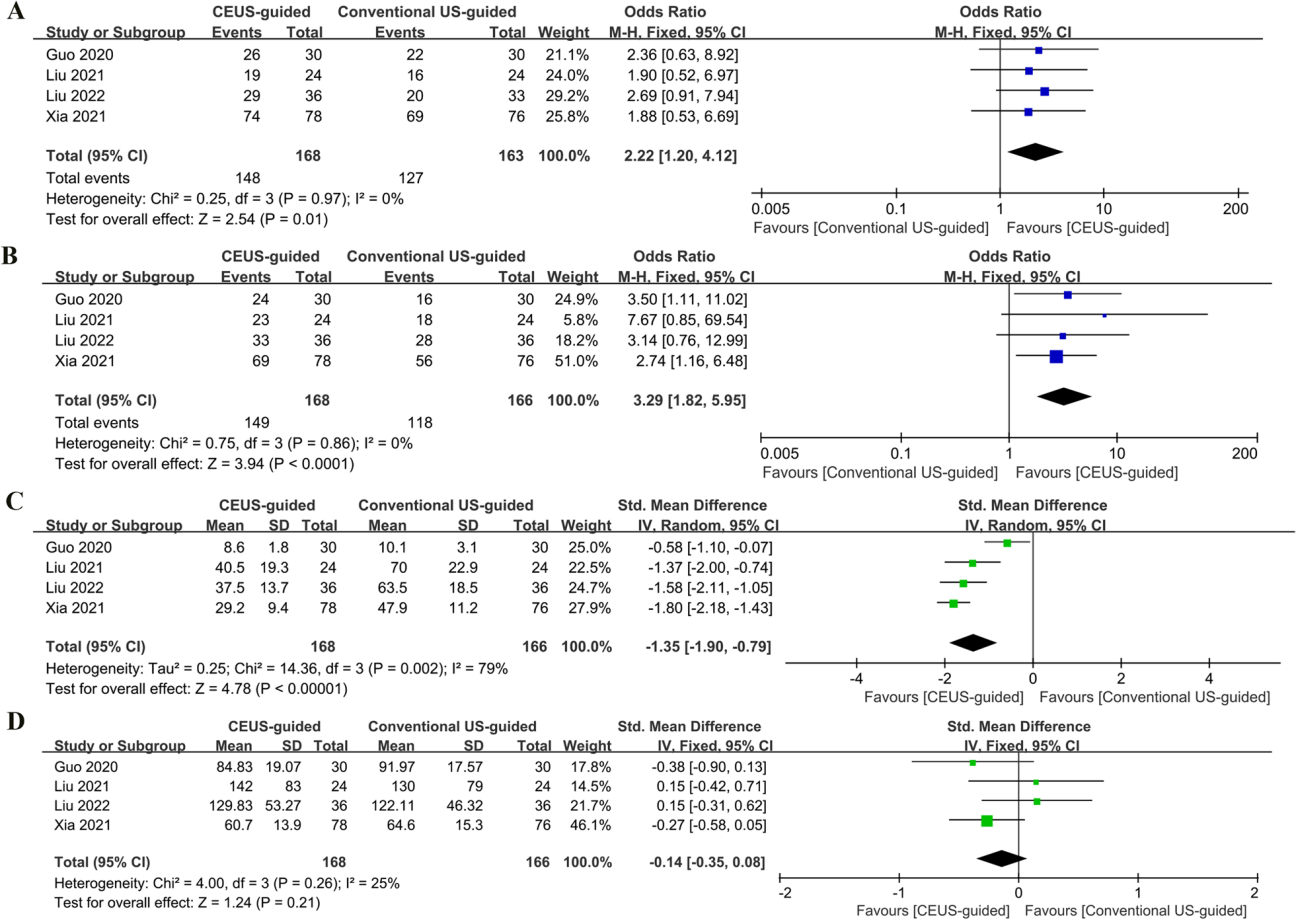
Forest plots of the perioperative outcomes: **A** stone-free rate; **B** single needle puncture success rate; **C** puncture time; **D** operation time

Based on a cumulative analysis of four studies [8–10, 18], CEUS-guided PCNL demonstrated a higher SFR (OR:2.22; 95% CI 1.2 to 4.12; P = 0.01; Fig. 3A), greater single-needle puncture success rate (OR:3.29; 95%CI 1.82 to 5.95; P < 0.0001; Fig. 3B), and shorter puncture time (SMD: − 1.35; 95% CI − 1.9 to − 0.79; P < 0.00001; Fig. 3C) as compared with conventional US-guided PCNL, with no significant difference in the operation time (SMD: − 0.14; 95% CI − 0.35 to 0.08; P = 0.21; Fig. 3D).

**Fig. 4:**
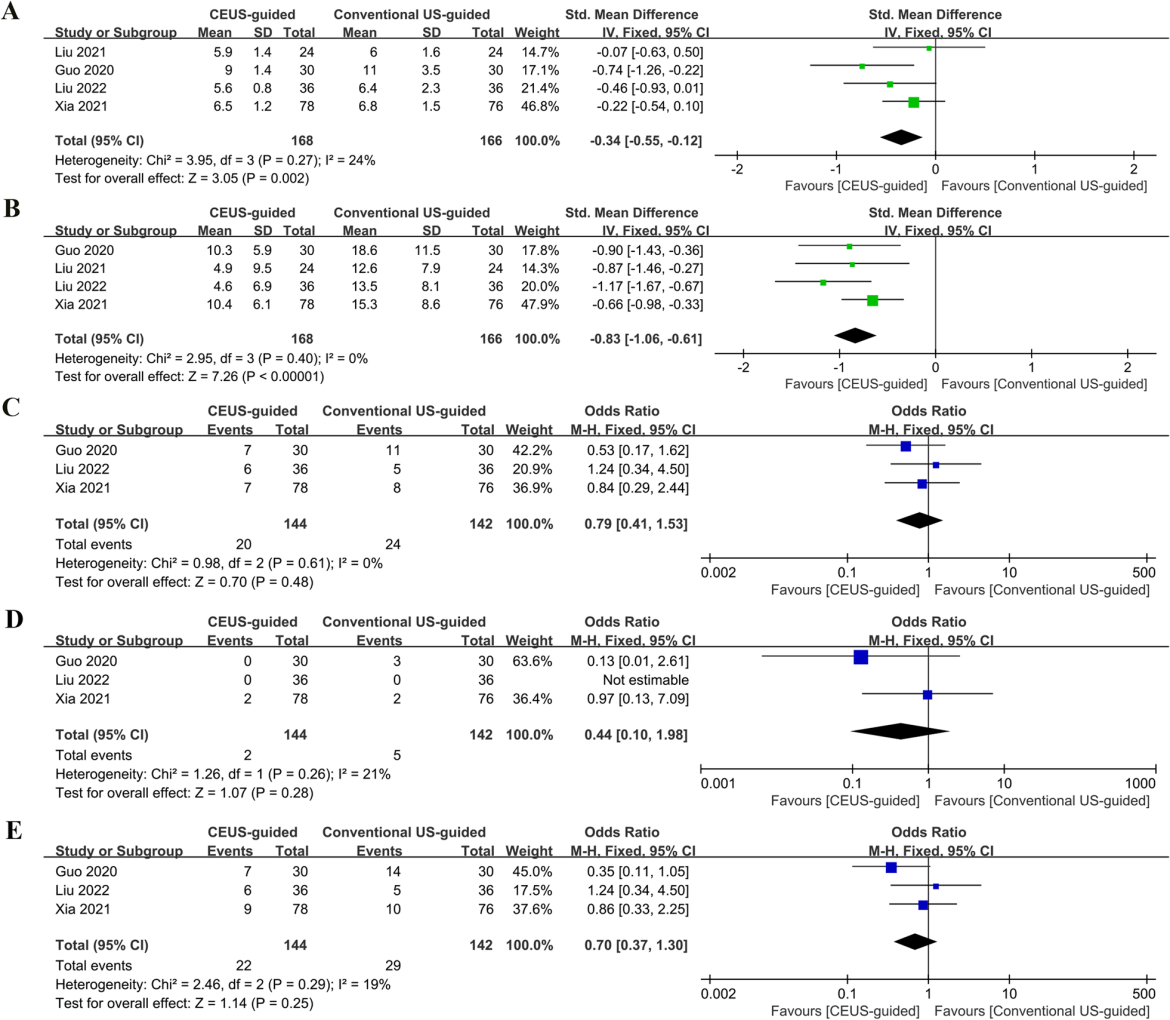
Forest plots of perioperative outcomes: **A** hospital stay; **B** hemoglobin loss; **C** minor complications; **D** major complications **E** overall complications

The hospital stay was significantly lower for CEUS-guided PCNL than for conventional US-guided PCNL (SMD: − 0.34; 95% CI − 0.55 to − 0.12; p = 0.002; Fig. 4A) [8–10, 18]. In addition, CEUS-guided PCNL reported lower hemoglobin loss than conventional US-guided PCNL (SMD: − 0.83; 95% CI − 1.06 to − 0.61; p < 0.00001; Fig. 4B) [8–10, 18].

There were no statistically significant differences between the two groups in terms of the rates of minor complications (Clavien-Dindo classification < grade 3) (OR: 0.79; 95% CI 0.41 to 1.53; p = 0.48; Fig. 4C) and major (Clavien-Dindo classification ≥ grade 3) complication rate (OR: 0.44; 95% CI 0.1 to 1.98; p = 0.28; Fig. 4D). The overall complication rates were 15.2% and 20.4% for the CEUS-guided PCNL and conventional US-guided PCNL groups, respectively (OR: 0.7; 95% CI 0.37 to 1.3; p = 0.25; Fig. 4E) [8–10].

### Heterogeneity

Most of the results showed low heterogeneity. However, the puncture time inevitably showed high heterogeneity (I^2^ = 79%). Of course, the bias introduced by small-sample studies cannot be ignored [19].

### Sensitivity analysis and publication bias

**Fig. 5:**
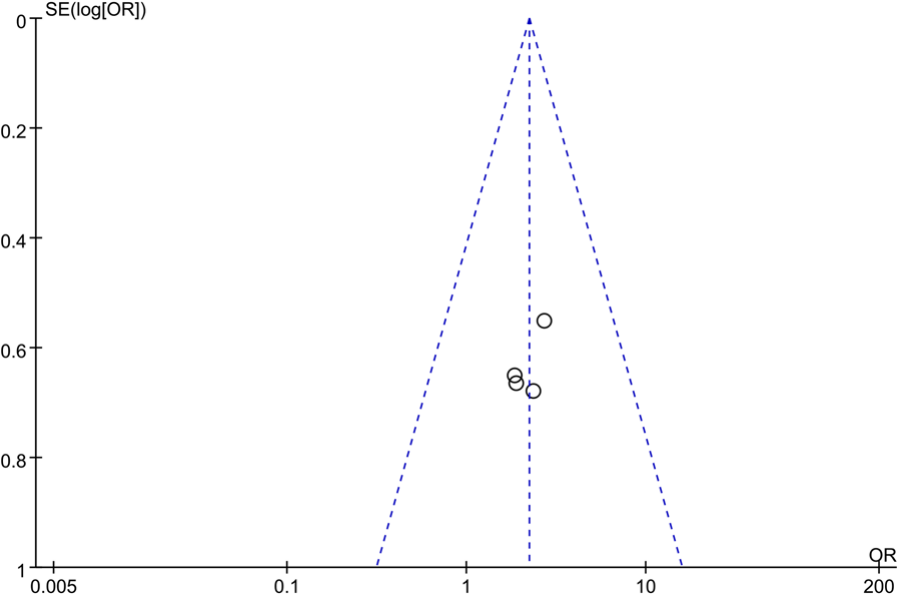
Funnel plot for the stone-free rate

We performed sensitivity analyses using the leave-one-out methods to test the data robustness and explore potential sources of high heterogeneity in the outcome variables. however, sensitivity analysis could not be performed when comparing three or fewer studies. Notably, sensitivity analysis significantly reduced the heterogeneity (I^2^ = 0; p < 0.0001) in the cumulative analysis of the puncture time when the study by Guo et al. was excluded [8]. Overall, no significant change was observed in the pooled SMDs and ORs, unequivocally confirming the validity of our results. Although only four studies were included, the funnel plot was not significantly perceived to be asymmetric visually (Fig. 5).

## Discussion

In recent years, the use of CEUS in urology has become more widespread, narrowing the gaps caused by magnetic resonance imaging and computed tomography in the diagnosis of tumors, vesicoureteral reflux, and cysts [20]. We were curious if CEUS-guided could improve the accuracy and visibility of percutaneous renal puncture in patients with no apparent hydronephrosis.

To the best of our knowledge, this is the first meta-analysis that is based on four RCTs. We reached a compelling conclusion by comparing the effectiveness and safety of CEUS-guided PCNL with conventional US-guided PCNL through meta-analysis. Although many studies had small sample numbers and low methodological quality, the analysis of the pooled data showed that the CEUS-guided PCNL group performed better than the conventional US-guided PCNL group in terms of the SFR, single-needle puncture success rate, puncture time, hospital stay and blood loss.

Access to the renal pelvic system is the fundamental and critical phase of PCNL. Successful establishment of the working channel at the first renal puncture is important as it helps reduce complications and improve stone clearance. The most frequent procedures used for this purpose include ultrasonography or fluoroscopic guidance. The number of passes with a needle and the average time for PCN varies depending on the guiding technique and severity of hydronephrosis. Moreover, the number of needle passes is positively correlated with the needle puncture time.

By contrast, as reported by Egilmez et al. [21], more punctures are needed in patients without hydronephrosis. Three CT-guided punctures were performed in patients with grade 0–1, 2, and 3 hydronephrosis 68%, 10%, and 0% of the time, respectively. If the degree of hydronephrosis was lowered from grade 3 to 0–1, the median duration increased from 14 to 20 min. However, Yagci et al. [22] showed that if saline was retrogradely injected to extend the renal collecting system, the expansion barely persisted for 15 min and quickly returned to baseline after 30 min. High pressure in the renal collecting system may result in lymphatic reflux of the renal pelvis and systemic absorption of germs or endotoxins, increasing the risk of fever and diseases.

Line with the injection of sulfur hexafluoride microbubble ultrasound imaging for the percutaneous renal puncture process more visual and simplified. The positioning of the puncture point and the puncture direction is more accurate because ultrasound imaging can obtain a kidney image similar to an X-ray angiography; this helps fully observe the renal collecting system, and quickly locate the ideal calyces.

The renal calyx is the optimal location for renal puncture because it causes only minor vascular damage and provides optimal access for stone removal. According to multivariate logistic regression analysis, the lack of hydronephrosis is a significant risk factor for blood transfusion in traditional PCNL [23]. The whole renal collecting system may be observed by using retrograde US contrast injection, much like in iodinated contrast images. Choosing the best calyx puncture site will avoid the establishment of dual channels, which will contribute to increasing the stone clearance rate. The peripelvic fat and prone position are challenging to target, and the absence of distention makes it difficult to navigate a stable guidewire into the renal pelvis and down the ureter [24]. Supine punctures are suitable for patients with heart and lung conditions or bone deformities. Yang et al. demonstrated that UG-PCNL can be performed on in the supine position, without sacrificing its effectiveness [25]. Liu's findings suggest that the flank position of PCNL guided by CEUS had a similar SFR and overall complication rate to PCNL performed in the prone position in patients without hydronephrosis [9]. Because of the intuitive nature of CEUS, novices may easily comprehend and master the percutaneous renal puncture procedure, resulting in a decreased incidence of related problems.

The complications of PCNL include bleeding, infection, urine leakage, and peripheral organ injury. The modified Clavien scale was used to grade postoperative problems. No difference was found between the two methods. perhaps owing to the sample sizes and follow-up times. When all the reported problems for patients receiving PCNL with or without hydronephrosis were considered, overall complications were found to occur in approximately 7.6 to 10.2% of the patients. According to an earlier study, the total complication rate for PCN was six times higher in patients with a non-dilated collecting system than in those with a dilated collecting system [26]. Cui et al. [27] were the first to report the use of CEUS to direct PCN. CEUS was found to be advantageous for guiding PCN and minimizing catheter-related problems. Li et al. [28] performed 132 non-dilated PCNLs for kidney stones, and three cases required blood transfusion. Liu et al. [7] demonstrated that hematuria was the leading cause of mild problems after PCN. We hypothesize that this was caused by injury to the peripheral renal vasculature during inadequate puncture of the minor renal calyx as a result of limited visibility.

### Limitations

Our meta-analysis has the following limitations: (1) Due to the limited number of randomized controlled trials (RCTs) and the short follow-up period, only four studies were included, and caution is necessary when interpreting the results due to the potential errors that may arise from small sample sizes. (2) the included studies were all conducted in Chinese regional populations, and differences in the regional and population characteristics cannot be ignored. and (3) the complexity of the kidney stones, the surgeon’s proficiency level, and other conditions affecting the operations may have influenced our findings.

## Conclusions

Taken together, CEUS-guided PCNL may improve the effectiveness of PCNL in patients with no apparent hydronephrosis. CEUS-guided PCNL benefited the patients in terms of the SFR, single-needle puncture success rate, puncture time, hospital stay and hemoglobin loss. However, future studies with a larger sample size and comparisons with other procedures are warranted.

## Supplementary Information

**Additional file 1.** PRISMA_2020_checklist.

## Data Availability

If necessary, the corresponding raw data can be provided by the corresponding author.

## Abbreviations

SMD: Standard mean difference
OR: Odds ratio
CI: Confidence intervals
RCT: Randomized controlled trial
US: Ultrasound
CEUS: Contrast-enhanced ultrasound
PCNL: Percutaneous nephrolithotomy
PRISMA: The Standards of Preferred Reporting Items for Systematic Reviews and Meta-Analyses
SFR: Stone-free rate
BMI: Body mass index
LE: Level of evidence
